# Evolution and Structural Analyses of *Glossina morsitans* (Diptera; Glossinidae) Tetraspanins

**DOI:** 10.3390/insects5040885

**Published:** 2014-11-12

**Authors:** Edwin K. Murungi, Henry M. Kariithi, Vincent Adunga, Meshack Obonyo, Alan Christoffels

**Affiliations:** 1 South African National Bioinformatics Institute (SANBI), University of the Western Cape, Private Bag X79, Bellville, Cape Town 7535, South Africa; E-Mail: alan.christoffels@uwc.ac.za; 2 Biotechnology Research Institute, Kenya Agricultural and Livestock Research Organization (KALRO), P.O. Box 57811, Kaptagat Rd, Nairobi 00200, Kenya; 3 Laboratory of Virology, Wageningen University, Droevendaalsesteeg 1, Wageningen 6708 PB, The Netherlands; 4 Department of Biochemistry and Molecular Biology, Egerton University, P.O. Box 536, Egerton 20115, Kenya; E-Mails: vowino@gmail.com (V.A.); obonyom@gmail.com (M.O.)

**Keywords:** tetraspanins, GmTsp, LEL, CD63, *Glossina morsitans*, *Trypanosoma*, phylogenetics, modeling, positive selection

## Abstract

Tetraspanins are important conserved integral membrane proteins expressed in many organisms. Although there is limited knowledge about the full repertoire, evolution and structural characteristics of individual members in various organisms, data obtained so far show that tetraspanins play major roles in membrane biology, visual processing, memory, olfactory signal processing, and mechanosensory antennal inputs. Thus, these proteins are potential targets for control of insect pests. Here, we report that the genome of the tsetse fly, *Glossina morsitans* (Diptera: Glossinidae) encodes at least seventeen tetraspanins (GmTsps), all containing the signature features found in the tetraspanin superfamily members. Whereas six of the GmTsps have been previously reported, eleven could be classified as novel because their amino acid sequences do not map to characterized tetraspanins in the available protein data bases. We present a model of the GmTsps by using GmTsp42Ed, whose presence and expression has been recently detected by transcriptomics and proteomics analyses of *G. morsitans*. Phylogenetically, the identified GmTsps segregate into three major clusters. Structurally, the GmTsps are largely similar to vertebrate tetraspanins. In view of the exploitation of tetraspanins by organisms for survival, these proteins could be targeted using specific antibodies, recombinant large extracellular loop (LEL) domains, small-molecule mimetics and siRNAs as potential novel and efficacious putative targets to combat African trypanosomiasis by killing the tsetse fly vector.

## 1. Introduction

Tsetse flies (genus *Glossina*) are vectors of the unicellular flagellated trypanosome parasites (genus *Trypanosoma*) that cause African trypanosomiases, a group of debilitating zoonotic neglected tropical diseases (NTDs) commonly referred to as sleeping sickness in humans and *nagana* in cattle [1]. African trypanosomiases have been described as one of the “root causes of hunger and poverty” in sub-Saharan Africa [2]. Left untreated, these diseases can be fatal, with fatalities differing from one group of trypanosome to another [3,4]. The diseases are difficult to treat, and there are no efficacious vaccines. None of the available trypanocidal drugs are ideal: the most widely used drug, melarsoprol, is toxic and up to 10% of the patients die from the treatment itself [5,6]. Besides, the treatment schedules for these drugs are prolonged, excruciatingly painful, and requires continuous hospitalization [7]. Therefore, the control of the disease vector (tsetse) is of critical importance, and probably represents the most sustainable trypanosomiases control method. Vector control using tsetse fly insecticide-impregnated traps [8], application of broad-spectrum insecticides [9], live baits [10], and the mating of virgin wild-type females with sexually-sterilized males [11] are some of the strategies applied to combat African trypanosomiasis. Although effective, some of these methods suffer several drawbacks such as increasing drug resistance/counterfeits [12,13], drug toxicity [5,6], and various environmental concerns such as loss of biodiversity and uncertainties on the fate of non-target organisms [14]. As such, identification of novel molecular targets that could be disrupted in the insect vector may provide new approaches of combating the diseases. One of the potential molecular targets is the tetraspanins superfamily (transmembrane 4 superfamily; TM4SF), a growing protein family of evolutionarily conserved integral membrane/surface proteins expressed in a wide range of multi-cellular organisms [15,16]. Available data suggest that pathogens (viruses, intracellular bacteria, and parasites) can “hijack” tetraspanins to gain entry into cells, for cytoplasmic trafficking after infection, and for final egress [17].

Tetraspanins are small (200–350 amino acid residues) type III surface glycoproteins with well defined structural motifs: short intracellular N- and C-termini connected by four transmembrane domains (TM1-TM4) with several conserved polar residues, which are interconnected by one small extracellular loop (SEL) and one large extracellular loop (LEL) [18,19]. The SEL domain contains 20–28 amino acid residues, while the LEL domain is made up of 76–131 amino acid residues [20]. The LEL domain is located between TM3 and TM4 and characteristically contains four to eight invariant conserved cysteine residues, two of which define a conserved Cys-Cys-Gly (CCG) motif and form intramolecular disulfide bonds crucial for structural integrity and functional specificities to tetraspanins [21,22,23]. More than 50% of tetraspanins also contain a Pro-x-x-Cys-Cys (PxxCC) motif, where “x” is any amino acid [24]. Tetraspanin proteins are synthesized in the endoplasmic reticulum (ER), and after palmitoylation, these proteins often form homodimers, which are subsequently transported to the cell surface to function as building blocks of large integrated signaling complexes or tetraspanin-enriched microdomains (TEMs) [25]. A combination of the above-mentioned features distinguishes tetraspanins from other four TM-domain proteins.

Tetraspanins are expressed in various cell types, and are implicated in a multitude of biological processes including signaling, cell adhesion, intracellular trafficking, and pathogen infections [20,26,27,28]. They facilitate these processes by a flare of rather promiscuous and unique associations with one another, and with a variety of non-tetraspanin integral macromolecules such as integrins [29], growth factor receptors/co-receptors, proteoglycans, complement-regulatory proteins, uroplakins, rhodopsin, members of the immunoglobulin superfamily, and others [30,31,32]. Via these interactions, tetraspanins form the TEM networks. The complexes arising from these protein-protein interactions have been proposed to play co-stimulatory roles in the activation of intracellular signaling pathways such as the N-terminal Jun Kinase pathway [33]. In animals, tetraspanins function as organizers of membrane signaling complexes, including cytoplasmic enzymes such as protein kinase C (PKC), phosphatidylinositol-4-kinase, and membrane components such as integrins and lipids [24,34]. Although the formation of the tetraspanins-PKC complex is independent of integrins, tetraspanins act as linker molecules by recruiting PKC into proximity with specific integrins.

Despite their biological importance, most tetraspanins have not been functionally explored because of their subtle and overlapping roles [35]. However, available data provide sufficient evidence that tetraspanins play important roles during bacterial [36], protozoal [37], and entomopathogenic fungal [38] infections. Since pathogens often “hijack” their hosts’ tetraspanin-organized natural cell processes (e.g., adhesion, internalization, vesicle trafficking, *etc.*) [39,40], the pathogen-tetraspaspanin interaction potentially offers novel therapeutic strategies against pathogenesis. Potentially, various *Trypanosoma* lifecycle stages could be targeted by tetraspanin-specific agents, such that anti-trypanocidal agents could be delivered to the parasite-containing vesicles. The advantage of such an approach is that tetraspanins are host-derived, implying that the possibility of the parasite developing resistance is much lower as opposed to the application of conventional trypanocidal drugs. Interrupting cellular processes that trypanosomes depend on for infection, multiplication/proliferation, and dissemination could greatly complement other available vector control strategies against African trypanosomiasis.

Due to their documented roles in pathogen-host interactions, tetraspanins are potential targets to control transmission of disease-causing parasites by insect vectors. For instance, insect tetraspanins could be targets for specific antibodies, recombinant LEL domains, small-molecule mimetics and siRNAs as potential strategies to combat diseases, in the interest of this work, African trypanosomiasis. Towards this end, we characterized the putative *Glossina morsitans* tetraspanins (abbreviated in this paper as GmTsps), and modeled for these proteins by using GmTsp42Ed. It is our opinion that the identification of tetraspanins will uncover efficacious novel insecticide targets against the tsetse fly.

## 2. Experimental

### 2.1. Sequential Retrieval, Ortholog Search and Bioinformatic Analyses of Tetraspanins

The tetraspanin gene sequences were retrieved from the UniProt database [41] for *Drosophila melanogaster* and *Musca domestica*, and from the VectorBase [42] for *Glossina morsitans*. To identify genes encoding tetraspanins in *G. morsitans*, the retrieved *D. melanogaster* annotated tetraspanins were used to construct a query protein set. BLASTp [43] was then used to search for all tetraspanin genes using a threshold setting as E-values ≤ 1e-4. The putative *G. morsitans* tetraspanins obtained were then used to construct a Hidden Markov model (HMM) to exhaustively search the *G. morsitans* genome. To avoid false-positive hits that commonly arise during automated searches, the presence of the conserved LEL domain in each putative tetraspanin obtained was ascertained using InterPro version 48.0 [44]. The new nucleotide and deduced amino acid sequences of tetraspanins were analyzed from the recently published *G. morsitans* genomic data sets [45]. Transmembrane domains in the candidate sequences were then predicted using SMART [46] and THMHMM server v. 2.0 [47]. The TM helices of the candidate tetraspanins and their membrane-spanning segments were further discriminated using SOSUI [48], and the hydrophobicity quality of the protein sequences was predicted using ProtScale at ExPASy bioinformatics resource portal [49]. Further analyses of the identified *G. morsitans* putative tetraspanins were annotated using the Blast2GO v. 2.7.2 [50]. To investigate whether the retrieved *G. morsitans* putative tetraspanins are palmitoylated, we used the clustering and scoring strategy (CSS) algorithm (at high threshold), CSS-Palm v. 4.0, which predicts the likelihood of palmitoylation within inputted amino acid sequences [51].

### 2.2. Multiple Sequence Alignment and Phylogenetic Analysis

Putative tetraspanins sequences were aligned using MUSCLE [52] with default settings. Sites with alignment ambiguities were excluded manually using Jalview [53]. Maximum likelihood (ML) and Bayesian phylogenetic searches were performed using the Le and Gascuel (LG) model of amino acid substitution [54]. The model of sequence evolution prior to each analysis was determined using Prot-Test v. 3.2.1 [55]. The ML analysis was performed using the program PhyML v. 3.0 [56] with the following parameters: substitution model = LG; prop_invar = 0.0; gamma = empirical; nb_subst_cat = 4. Bootstraps for the ML were generated using 100 replicates of bootstrapping. Support for the nodes was assessed with 100 bootstrap replicates. Bayesian inference analysis was conducted with MrBayes v. 3.2 [57]. Ten million generations of MCMC simulation were used along with a burnin of 10,000 generations. This number of MCMC generations allowed for convergence of simulation chains and reduction of split frequencies to an acceptable level. Phylogenetic trees were built using the ML method and rendered using the interactive Tree Of Life (iTOL) v. 2.2.2 [58].

### 2.3. Positive Selection Analysis

Patterns of sequence change using nonsynonymous/synonymous (*d*_N_*/d*_S_) rate ratios were performed using five methods for detecting positive selection available from the DATAMONKEY [59] web server [59]. The five methods used in this study were: Single Likelihood Ancestor Counting model (SLAC), the Fixed Effect Likelihood model (FEL), the Random Effect Likelihood model (REL), the Mixed Effects Model of Evolution (MEME) and the Fast Unbiased Bayesian AppRoximation (FUBAR) [60,61,62]. For these analyses, the best fitting nucleotide substitution model was determined through the automatic model selection tool available on the DATAMONKEY server. Prior to running the analyses, the dataset was screened for recombination using GARD [63]. Recombination can contribute to false inference of positive selection, causing a high rate of false positive detection. No evidence of recombination was found.

### 2.4. Protein Modeling

To obtain the appropriate template for homology modeling, the putative tetraspanin sequences were threaded through protein homology/analogy recognition engine platform (PHYRE) v 2.0 [64]. Homology models of tetraspanins were constructed using the program MODELLER v. 9.13 [65]. An alignment of query sequences with template proteins was used as input for modeling MODELLER, and 100 comparative models were generated for each putative tetraspanin sequence. The models were subsequently validated using MODELLER objective function and DOPE score, statistical parameters for the assessment of the model was done using the standard Modeler energy function. The model quality and accuracy were stereochemically ascertained by using the protein structure and verification tool, PROCHECK v. 3.5.4 [66,67]. The quality of the modeled structures were also validated by other structure verification servers such as VERIFY 3D (to analyze the compatibility of the 3D structure with the amino acid sequences [68]), and ERRAT (to statistically analyze non-bonded interactions between different atoms, whereby higher scores are indicative of higher quality [69]). The tetraspanin domains were mined from the Conserved Domain Database [70].

## 3. Results and Discussion

Tetraspanins are an evolutionarily conserved protein superfamily that have been investigated for their potential functions as “molecular facilitators” of cell growth, motility, signal transduction, and host-pathogen interactions [15,71]. The present study was conceived from the exhaustive collation of the available data reporting the existence of tetraspanin superfamily members in various organisms whose genomes have been sequenced [72]. Of particular interest in this study is the possibility that given the important roles that tetraspanins play, their disruption would putatively jettison the parasite transmitting cycle of *G. morsitans*. These proteins could therefore be potential targets aimed at mitigating the vectorial ability of *G. morsitans* in the control of African trypanosomiasis. Several studies have demonstrated the critical roles of tetraspanins during parasite infection in insect vectors. For instance, Jaramillo-Gutierrez *et al.* [73] showed that a knockdown of tetraspanins enhanced *Plasmodium* parasite infection in anopheline mosquitoes, potentially via blockade of immune cascades mediated by these proteins. With the recent availability of the *G. morsitans* genome sequence [45], it is interesting to identify and analyze tetraspanins in this insect. Therefore, here, we identified, structurally characterized and analyzed the evolution of tetraspanins in *G. morsitans in silico*.

### 3.1. Glossina morsitans Putative Tetraspanins

**Figure 1:**
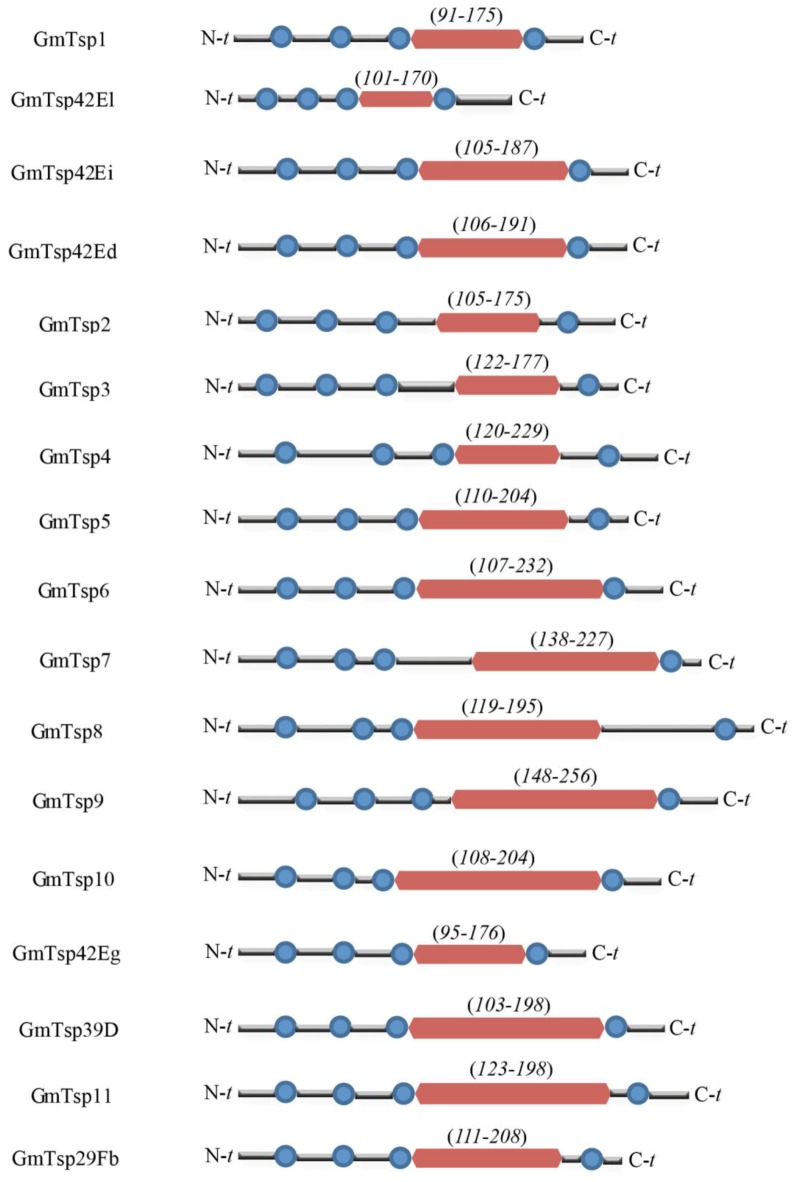
Domain architecture of *Glossina morsitans* putative tetraspanins. Predicted domain structures of the seventeen *G. morsitans* are indicated. Transmembrane and large extracellular domain (LEL) domains are depicted as blue round circles and magenta rectangular blocks, respectively. The numbers in the parentheses indicate the coordinates (amino acid residues) of the LEL For details of the coordinates of the TMs, compare this figure with data presented in Table 1 and in Figure A1. N-*t* and C-*t* represents the N- and C-termini of the GmTsp proteins, respectively.

**Figure A1:**
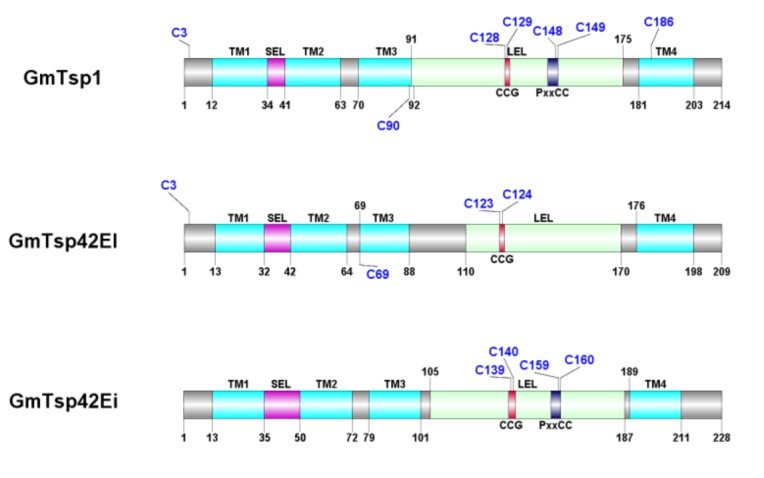

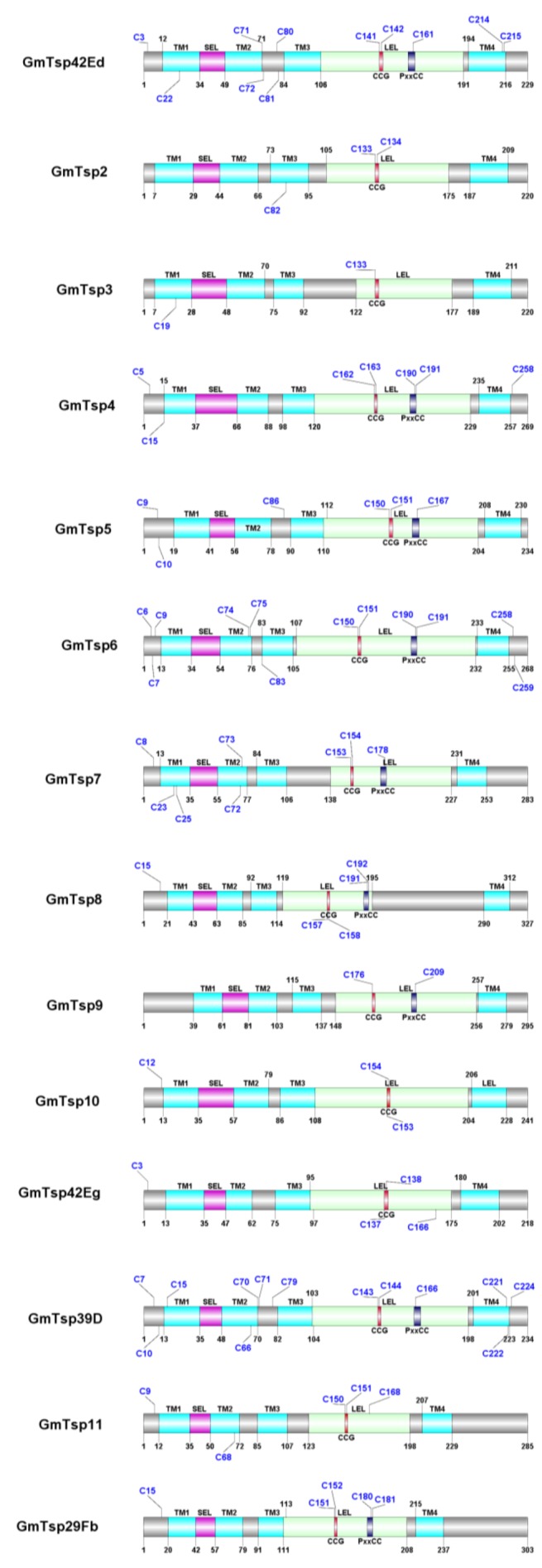
Distribution of the structural features of seventeen *G. morsitans* putative tetraspanins detected in this study: The start and end of the domains/motifs are indicated by the coordinates of the amino acid residues (shown with black numbers with vertical bars). The palmitoylation sites are shown in blue bold letters; the transmembrane domains(TM1–4) are depicted in light blue; the SEL motifs are in pink; the CCG domains are shown in red; the LEL motifs are shown in light green, while the PxxCC motifs are in dark blue. Notice that six of the GmTsps do not contain the PxxCC motif.

We identified and characterized a repertoire of tetraspanins in the genome of *Glossina morsitans* based on their definitive structural characteristics of the tetraspanin family of proteins. The presence of TM domains in the putative tetraspanins was determined using SMART and TMHMM 2.0 while the definitive extracellular domain was identified using InterPro. For the purposes of clarity in this study, we abbreviated the identified *G. morsitans* tetraspanins as “GmTsp”, an abbreviation used hereafter. Notably, the identified *G. morsitans* tetraspanins contained one LEL domain at the C-terminus, and four TM domains (Figure 1 and Table 1). We further evaluated the putative GmTsps by functional annotation using Blas2GO. Our analysis for the presence of the structural hallmarks of the tetraspanin protein family (see introduction) indicated that the *G. morsitans* genome contained twenty four Tsps (here, we do not show the data for all the twenty four Tsps). However, more stringent analysis (concentrating on the putative GmTsps that fulfill the requirement for four hydrophobic helices, SEL, LEL, presence of palmitoylation sites, and cytosolic orientation of the N- and C-terminal of the proteins) revealed that only seventeen of the putative GmTsps qualified as members of the tetraspanin superfamily. We confirmed that the N- and the C-terminal of the seventeen putative GmTsps are cytosolic with reference to the plasma membrane. Although we have not shown the data for all the putative GmTsps, we have shown the orientation of a representative of GmTsps using GmTsp42Ed in comparison with the human HsTspCD63 (see discussion later in this paper). Since the cytosolic orientation of these termini is important in cellular signaling, this result implies that the putative GmTsps are highly likely to have similar functions reported for other members of the tetraspanin superfamily. The presence of the structural hallmarks of the GmTsps is shown in Figure A1. Of the seventeen GmTsps, six were reported in the sialome [74] and transcriptome of *G. morsitans* [45], and their protein names and UniProt IDs are indicated in Table 1. The amino acid sequences of the remaining eleven of the seventeen GmTsps did not match to any sequences and/or information in the available protein databases other than in the VectorBase. These proteins appear as novel tetraspanins in the genome of *G. morsitans* because they only matched to EST sequences on VectorBase and no gene function for these sequences has been so far determined or inferred. For clarity, we arbitrarily named (and abbreviated) these eleven new tetraspanins as GmTsp 1 to 11 (see Table 1). Notably, all the identified GmTsps identified in this study fell within the size range (200–350) amino acid residues [16]) of the various tetraspanins reported to date.

**Figure 2:**
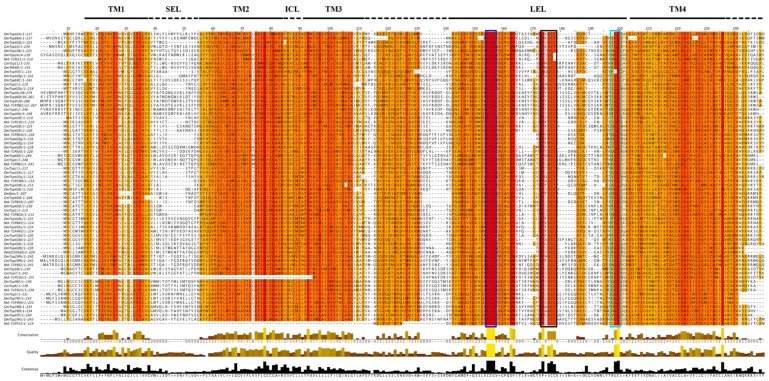
Alignment of the *G. morsitans* GmTsps: The shared secondary structural of the tetraspanins (TM 1–4; SEL, and LEL), the conserved CCG, PxxCC motif (boxed) and the terminal region rich in charged/polar amino acids are shown.

Alignment of *G. morsitans* tetraspanin amino acid sequences alongside homologs from the house fly, the fruit fly, and human, revealed that the GmTsps have the two highly conserved features of tetraspanins: (i) the LEL harboring the CCG motif; and (ii) the four TM domains with some well-conserved residues (Figure 2). Other features characteristic of tetraspanins, *i.e.*, SEL, ICL and C-terminal region rich in charged/polar amino acids were observed as evident in Figure 2. Further, in addition to the CCG motif, the other additional motifs, *i.e.*, PxxCC and EGC were also present in the GmTsps (Figure 2). Notably, of the seventeen putative GmTsps that we identified, 64.7% (*n* = 11) contained the additional PxxCC within the LEL domain (Figure A1), which is in agreement with the documented data that more the 50% of Tsps contain this motif [24]. Taken together, these findings show that *G. morsitans* tetraspanins have all the structural hallmarks of the typical tetraspanins found in other insects and humans, and suggest that the putative *G. morsitans* tetraspanins identified in this study could in general be classified as members of the tetraspanin superfamily of proteins.

**Table 1:** Domain characteristics of seventeen GmTsp proteins in the genome of *G. morsitans*. The coordinates of the large extracellular loop (LEL) and the four transmembrane domains (TM) are shown in columns 4 and 5.

Protein Name (Description *)	UniProt/VectorBase ID	Length [aa]	LEL Domain Coordinates	TM Domain Coordinates
GmTsp1 (Protein late bloomer-like)	GMOY003645-PA ^¥^	214	91–175	12–34, 41–63, 70–92, 181–203
GmTsp42El	D3TMF8	209	110–170	13–32, 42–64, 69-88, 176-198
GmTsp 42Ei	D3TL43	228	105–187	13-35, 50–72, 79–101, 189–211
GmTsp42Ed	D3TMA1	229	106–191	12–34, 49–71, 84–106, 194–216
GmTsp2 (Tetraspanin EC2/Peripherin)	GMOY003647-PA ^¥^	220	105–175	7–29, 44–66, 73–95, 187–209
GmTsp3 (Protein late bloomer-like)	GMOY003648-PA ^¥^	220	122–177	7–28, 48–70, 75–92, 189–211
GmTsp4 (Tetraspanin, isoform A)	GMOY003747-PA ^¥^	269	120–229	15–37, 66–88, 98–120, 235–257
GmTsp5 (CD151 antigen-like protein, isoform x2)	GMOY007608-PA ^¥^	234	110–204	19–41, 56–78, 90–112, 208–230
GmTsp6 (Tetraspanin family integral membrane protein, isoform B)	D3TLU9	268	107–232	12–34, 54–76, 83–105, 233–255
GmTsp7 (Tetraspanin, isoform C)	GMOY010508-PA ^¥^	283	138–227	13–35, 55–77, 84–106, 231–253
GmTsp8 (Tetraspanin-5-like, isoform x1)	GMOY004352-PA ^¥^	327	119–195	21–43, 63–85, 92–114, 290–312
GmTsp9 (Tetraspanin-33-like, isoform x1)	GMOY011478-PA ^¥^	295	148–256	39–61, 81–103, 115–137, 257–279
GmTsp10 (CD63 antigen-like protein)	GMOY000619-PA ^¥^	241	108–204	13–35, 57–79, 86–108, 206–228
GmTsp42Eg	D3TQ76	218	95–176	13–35, 40–62, 75–97, 180–202
GmTsp39D	GMOY010261-PA ^¥^	234	103–198	13–35, 48–70, 82–104, 201–223
GmTsp11 (Transmembrane 4 protein, isoform C)	GMOY009229-PA ^¥^	285	123–198	12–35, 50–72, 85–107, 207–229
GmTsp29Fb	D3TQ22	303	111–208	20–42, 57–79, 91–113, 215–237

* The description of the proteins were performed using Blast2GO v 2.7.2 [50]; ^¥^ These proteins did not match to sequences and/or information in biological databases other than the VectorBase. The apparently new *G. morsitans* tetraspanins have been arbitrarily abbreviated as GmTsp 1 to 11.

Generally, protein palmitoylation is a special class of covalent post-translational modification which reversibly mediates various cellular processes. In particular, palmitoylation ensures not only surface hydrophobicity and membrane affinity, but also plays important roles in modulating the trafficking, stability, sorting, protein-protein and protein-lipid interactions of the modified proteins [75,76]. Various studies have demonstrated palmitoylation of tetraspanins on the cytoplasmic cysteine residues proximal to the plasma membranes contributes to the organization of these proteins into TEMs [77,78,79], underpinning the role of palmitoylation in functional integrity of tetraspanins. The results of palmitoylation prediction of GmTsps revealed that, as typical of tetraspanins [77,78,79], the predicted palmitoylation sites in the GmTsps involve multiple Cysteine residues adjacent to the borders between the cytoplasmic and TM domains (compare data presented in Figure A1 and Table 1). It should however be noted that the palmitoylation sites in the GmTsps shown in the Figure A1 are predicted, implying that some of these sites may not be truly palmitoylated, especially those that are predicted to be on the cysteine residues in the CCG and the PxxCC motifs.

### 3.2. Evolutionary and Phylogenetic Analysis

**Figure 3:**
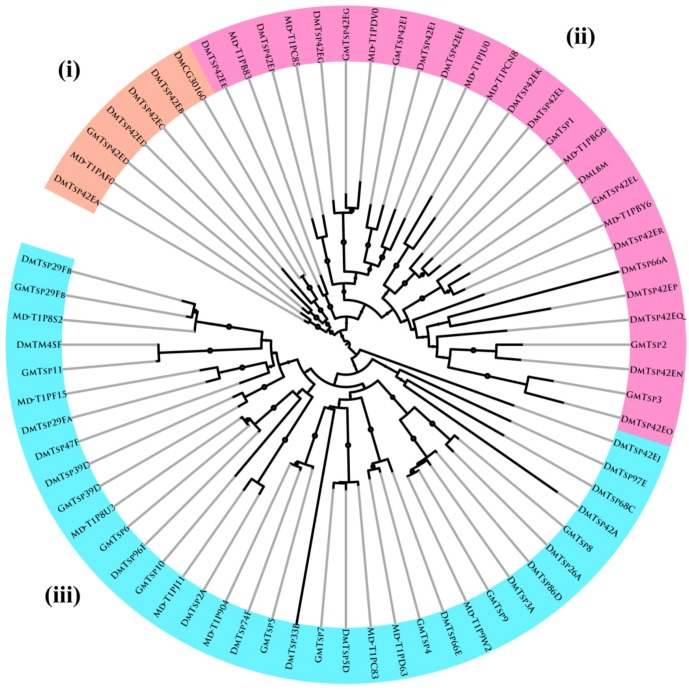
Phylogenetic analysis of tetraspanin homologs based on amino acid sequences: The phylogenetic tree shows clustering of the Tsps into three main clades and nine sub-clades, which display 1:1:1 orthologous relationships of these proteins. The phylogenetic tree was constructed using the ML method implemented in PhyML [81] using the LG model of amino acid substitution [54]. The seventeen putative tsetse fly (*G. morsitans*) tetraspanins identified in this study (see Table 1) were phylogenetically compared to homologs in the fruit fly (*D. melanogaster*) and housefly (*M. domestica*). Nodes with bootstrap support values >80% are marked with solid circles. Gm, *Glossina morsitans*; Dm, *Drosophila melanogaster*; Md, *Musca domestica*. **Note:** for the abbreviation of the Tsps in this figure, all letters are in upper case; the letters written in lower case in the main text are shown here in decreased font size.

To explore phylogenetic relationship among tetraspanins in different insect species, a phylogenetic tree was constructed including tetraspanins from *D. melanogaster* [56] and *M. domestica*. We initially included human (*H. sapiens*) tetraspanins in the evolutionary analysis to determine if they co-evolved with the insects’ tetraspanins. Tetraspanins were originally identified in humans as putative mediators of tumor progression [80]. In this paper, we have not shown data on the analysis that included the human tetraspanins. The unrooted phylogenetic tree (Figure 3) was generated by the alignment of full-length protein sequences of 76 tetraspanin proteins and was inferred using both Bayesian inference and maximum likelihood (ML) methods. Tree nodes supported by high posterior probability and bootstrap values for the two methods were considered robust. A list of all amino acid sequences used for phylogenetic analysis is provided in Supplemental File S1.

As shown in Figure 3, three distinct clusters were observed: (i) A seven-member cluster consisting of one from *G. morsitans* (GmTsp42Ed), three from *D. melanogaster* (DmTsp42Ea, DmTsp42Ed, DmTsp42Ec, DmTsp42Eb and DmCG30160) and one from *M. domestica* (Md-T1PAF0); (ii) A twenty seven-member cluster with six *G. morsitans* (GmTsp42Eg, GmTsp42Ei, GmTsp1, GmTsp42EI, GmTsp2 and GmTsp3), fourteen *D. melanogaster* (DmTsp42Eo, DmTsp42En, DmTsp42Eq, DmTsp42Ep, DmTsp66A, DmTsp42Er, Dmlbm, DmTsp42El, DmTsp42Ek, DmTsp42Eh, DmTsp42Ei, DmTsp42Eg, DmTsp42Ef and DmTsp42Ee) and seven *M. domestica* (Md-TIPB83, Md-TIPC85, Md-TIPDV0, Md-TIPJU0, Md-TIPCN8, Md-TIPBG6 and Md-TIPBY6); (iii) A thirty five-member cluster made up of nine *G. morsitans* tetraspanins (GmTsp11, GmTsp39D, GmTsp6, GmTsp10, GmTsp5, GmTsp7, GmTsp4, GmTsp9 and GmTsp8), eighteen *D. melanogaster* (DmTsp29Fb, DmTM4SF, DmTsp29Fa, DmTsp47F, DmTsp39D, DmTsp96F, DmTsp2A, DmTsp74F, DmTsp33B, DmTsp5D, DmTsp66E, DmTsp3A, DmTsp86D, DmTsp26A, DmTsp42A, DmTsp68C, DmTsp97E and DmTsp42Ej) and eight *M. domestica* tetraspanins (Md-T1P8S2, Md-T1PF15, Md-T1P8U3, Md-T1PJ11, Md-T1P904, Md-T1PC83, Md-T1PD63 and Md-T1P9W2).

In general, the clustering pattern observed in Figure 3 is indicative of gene duplications, gene loss and species-specific expansion in the various clades, as evidenced by the sub-clades in the three major clades. Further, within the three main clades, there are nine sub-clades displaying 1:1:1 orthologous relationship. The conservation of tetraspanins in the three insect species points to a probable critical role for the gene. Only a few clades show evidence of putative species-specific expansion. Overall, in most (sub-) clades there is evidence of gene loss. Several orthologous relationships among the putative insect tetraspanins are evident. The wide presence of tetraspanins in almost all organisms indicates that they have experienced a long evolutionary history [82]. Based on the phylogenetic relationships and ancestral origin of tetraspanins, it is possible that they have evolved from a single or a few ancestral genes by gene duplication and divergence and this evolution is impacted by gene loss and positive selection on coding sequence [16,83].

### 3.3. Structural Analysis

**Figure 4:**
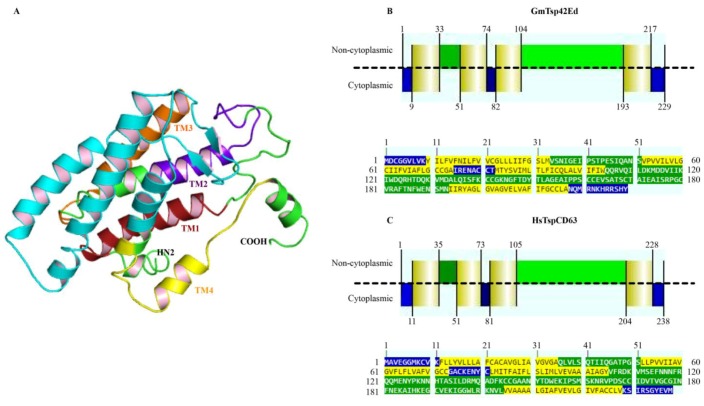
Cartoon representation of the modeled GmTsp42Ed structure: (**A**) 3-D representation of GmTsp42Ed model, in which the N- and the C-termini are shown as NH2 and COOH, respectively. The four transmembrane domains are color-coded as red, purple, orange and yellow for TM1 to TM4, respectively. The LEL domain which is located between TM3 and TM4 has three α-helices and two anti-parallel β-strands and is shown in cyan; (**B**,**C**) 2-D representation of the orientation of the cytoplasmic (shown in blue), non-cytoplasmic (small extracellular loop (SEL) and LEL domains) (shown in green), and TM1-4 helices (shown in yellow) of *G. morsitans* GmTsp42Ed and the human tetraspanin (HsTspCD63), respectively. The alignments of GmTsp42Ed and HsTspCD63 are shown at the bottom of the domain orientations. The dotted lines in Panels (**B**) and (**C**) represent a hypothetical cellular plasma membrane.

Of the seventeen *G. morsitans* putative tetraspanins identified in this study, we selected one, Tetraspanin 42Ed (GmTsp42Ed; UniProt ID # D3TMA1) for 3-D structural modeling. The main reason for selection of GmTsp42Ed was because, in addition to being detected at the transcript level [45,74], there is evidence that this protein is also expressed in the proteome of *Trypanosoma brucei-*infected *G. morsitans* salivary glands [84], indicative of a possible important role for this tetraspanin in *G. morsitans* vectorial ability. The modeling of GmTsp42Ed was performed using multiple templates (1G8Q, 4JKV, and 2M7Z) as identified in the threading sequence-structure engine PHYRE. Comparative homology modeling by satisfaction of spatial restraints using MODELLER resulted in a cylindrical and very compact 3-D model (Figure 4). The model has seven α-helices and two anti-parallel β-strands. Four helices (color coded in Figure 4) form the hydrophobic transmembrane domains while the remaining three are found in the extracellular LEL domain. Two anti-parallel β-strands are inserted between TM3 and the LEL helices resulting in a break in the continuity of the helical conformation. The anti-parallel β-strands probably form the non-conserved sub-domain of the LEL domain [85]. Both N-and C-terminal regions of GmTsp42Ed adopt somewhat ordered conformations. For the N-terminus, this is perhaps due to the palmitoylation of a cysteine 3 residue in this region. To further confirm whether the N- and C-termini of the GmTsp42Ed are oriented to the cytosolic side of the plasma membrane, we compared the amino acid sequence of this Tsp with the human HsTspCD63. The result of this comparative analysis revealed that, similar to the human homolog, the two termini of GmTsp42Ed are indeed cytosolic, and the degree of similarity between the two proteins was very high (Figure 4B,C).

**Figure 5:**
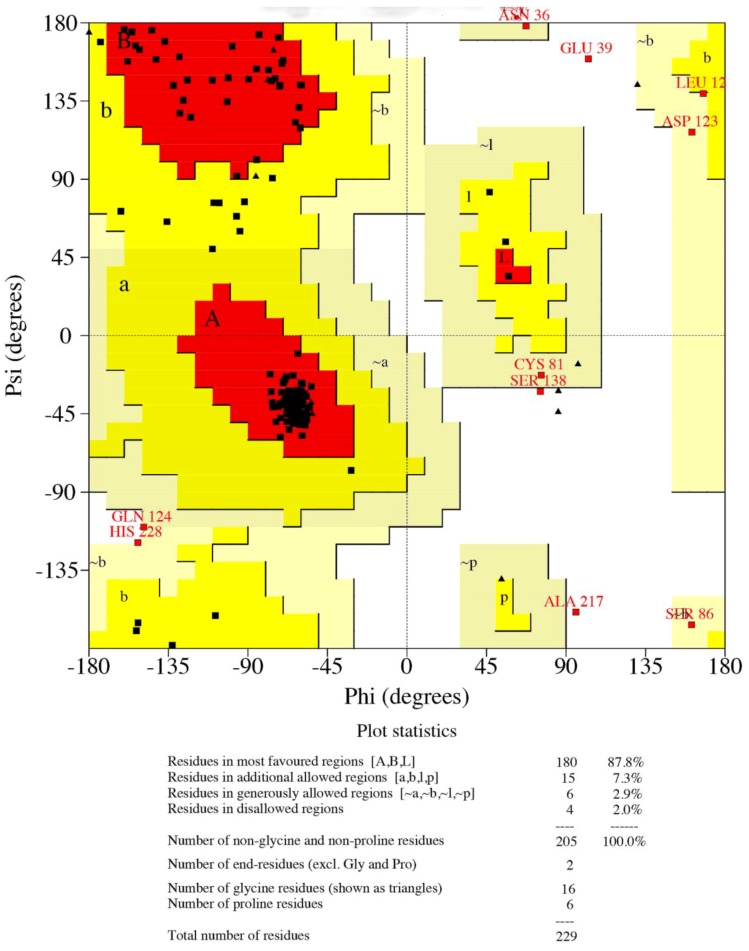
Ramachandran plots for GmTsp42Ed: The plot statistics are indicated for all the non-glycine and non-proline residues that fell within the favored regions. Shown in red, yellow, pale yellow, and white, respectively, are amino acid residues in most favored, additionally allowed, generously allowed, and disallowed regions. Based on the analysis of 118 structures of resolution of at least 2.0 Angstroms and R-factor no greater that 20%, a good model is expected to have ≥90% in the most favored regions. The plots were generated using PROCHECK v. 3.5.4 [66].

We stereochemically ascertained the quality and accuracy of the GmTsp42Ed using the PROCHECK protein structure validation and verification package. The Ramachandran plot obtained showed that 87.8%, 7.3%, 2.9%, and 2.0% of residues fell within the most favored regions, additionally favored regions, generously allowed regions and the disallowed regions, respectively (Figure 5). This makes a combined percentage of 98.0% of the residues in the favored and allowed regions for the model making it stereochemically robust [86]. Again, using GmTsp42Ed as a representative of *G. morsitans* tetraspanins, this result appears to confirm that in general, the GmTsps belong to the tetraspanin superfamily.

### 3.4. Positive Selection Analysis of G. morsitans Tetraspanins

Mutation and selection have different effects on nonsynonymous (amino-acid-replacement) and synonymous (silent) substitution rates (*d*_N_ and *d*_S_, respectively), and are therefore means of understanding the dynamics of molecular sequence evolution [87]. Models of variable *d*_N_/*d*_S_ ratios among sites provide important insights into the functional constraints at different amino acid sites, and are to detect sites under positive selection [88]. A *d*_N_/*d*_S_ >> 1.0 is considered as a convincing indicator of positive selection [89]. Analysis of selection pressures exerted on tetraspanins in *G. morsitans*, *D. melanogaster* and *M. domestica* revealed that several sites are under positive selection based on statistical significance tests as assessed by various models such as MEME, FEL, IFEL, REL and FUBAR (See Supplemental File S2). Unlike the other methods, MEME methodology can identify both episodic and persistent positive selection because it allows the distribution of the dN/dS ratio to vary from site to site and also from branch to branch at a site. Therefore, the additional positively selected codons identified by MEME and not by the other approaches, are likely to have been subject to episodes of positive selection. Overall, these results suggest that positive selection is an important contributor in the evolution of tetraspanins.

### 3.5. Targeting Tetraspanins as Potential Therapeutics against African Trypanosomes

Due to a lack or failure of conventional vaccines and drugs to combat insect-transmitted pathogens, the identification of novel therapeutic targets against infections is under intensive research. For African trypanosomiasis, there is need to address the problem of drug resistance and/or counterfeits [12,13], and a lack of efficacious vaccines [5,6] against this group of NTDs. An approach to this problem is to target cellular processes of the vector rather than targeting the *Trypanosoma* parasites. As described in Section 1 of this article, recent data have indicated that members of the tetraspanin superfamily have potential to provide such a novel approach. Specific tetraspanins family members have been shown to play roles in pathogen infections by selectively associating with the pathogens at multiple infection stages from the initial cellular attachment to the egress of mature pathogens (See Table 1 in Ref. [90] for tetraspanin family members with reported links to pathologies). Although the precise roles of TEMs during pathogen infections have not been extensively investigated, several studies have demonstrated that tetraspanin-based drugs (mimicry) can disrupt normal biological functions. For instance, inhibition of the binding of hepatitis C virus envelop glycoprotein EC to its receptor, CD81 [91]. Further, Spoden *et al*. [92] provided evidence that tetraspanin-specific antibodies and siRNAs inhibited both the cell entry and subsequent infection of the human papilloma virus type 16. Similar finding have been reported in the case of the infection of human immunodeficiency virus [93,94,95]. In addition to targeting host-encoded tetraspanins, other independent studies have explored the application of antibodies against recombinant TSPs from parasites. For instance, Tran *et al*. [96] demonstrated that recombinant antibodies to SmTSP-1/2 (cloned from the trematode *Schistosoma mansoni*) significantly reduced the parasite loads after challenge with *S. mansoni*. Silencing of *Sm-tsp-1* or *Sm-tsp-1* resulted in malformation of the parasite tegument and tetraspanins-depleted parasites were found to be defective in survival in the host [97], implying that Tsps have integral structural roles in the development and maintenance of the parasite's tegument. Similar to their application in the control of schistosomiasis, Tsps have the potential as vaccine candidates against filariasis. Although not yet fully worked out, Dakshinamoorthy *et al*. (2013) have recently demonstrated that antibodies to BmTSP LEL and WbTSP LEL (cloned from the nematodes *Brugia malayi* and *Wuchereria bancrofti*, respectively) conferred significant protection to mice that were challenged with filarial worms [98]. Future research in the case of *Trypanosoma* parasites should focus on the analysis of tetraspanins expressed by these parasites to explore their potential as vaccine candidates. Taken together, although we did not analyze *Trypanosoma*-specific tetraspanins in this article, the data presented in our study provide proof of principle that targeting tetraspanins, either vector- or parasite-encoded, is a promising strategy to inhibit specific stages of pathogen infection.

## 4. Conclusions

This work identified seventeen putative tetraspanins in the genome of the tsetse fly, *G. morsitans*, eleven of which appear to be novel as their proteins sequences did not match to any tetraspanin superfamily member either determined or inferred, rather, their sequences only matched to EST sequences in the VectorBase. The presence of the hallmark tetraspanin features, including the LEL domain harboring the CCG/PxxCC motifs, four TM domains with some well-conserved residues, the SEL, ICL and C-terminal region rich in charged/polar amino acids suggest that the putative *G. morsitans* tetraspanins identified in this study are *bona fide* members of the tetraspanin superfamily of proteins. However, there is need to experimentally validate the functionality of these tetraspanins in *Glossina*. Nevertheless, the results presented here constitute a platform for the expansion of future exploration into the biological roles of *G. morsitans* tetraspanins, and their potential as candidates for anti-*Trypanosoma* mitigation strategies. This is based on the fact that tetraspanins are not merely passive targets for pathogen infection, but appear to have more fundamental roles during initial attachment (cell-to-cell fusion) to host cells, cellular trafficking, and egress. This is a subject of our immediate further investigations for the identification and characterization of *Trypanosoma*-encoded tetraspanins as these are potential targets for the development of vaccines against the parasite.

## Supplementary Files

Supplementary File 1
