# Supplementary material for: Evolution and Structural Analyses of Glossina morsitans (Diptera; Glossinidae) Tetraspanins
Source: Insects. 2014 Nov 12;5(4):885–908. doi: 10.3390/insects5040885 (PMC4592607; doi:10.3390/insects5040885)
Supplement: Supplementary File 1 [file insects-05-00885-s001.pdf]

Supplementary Material

File S1. A list of all amino acid sequences used for the evolutionary and phylogenetic analysis of *G. morsitans* tetraspanins: The list contains seventeen, thirty-seven, and sixteen protein sequences from the genomes of *Glossina morsitans* (Gm), *Drosophila melanogaster* (Dm), and *Musca domestica* (Md), respectively.

>GmTsp1
MGCATITIKYLLYVFNTLWAIIGILLIILGGFGWDAMPRNYAIGVISLGGVILLIAFFGC
MGAARESARSLWTHAITMLVMIVLVVVMICFNTRDVFKKYALQEVNDFWQKEITQPGSMD
NMQIVYACCGRDSAEDYVNIGRSPPSSCCKDSNCINPLNVYITGCITKVEAAFADEVLTT
QICEWCLLGVSLVILLLATALAIHYTNQKRRFGY

>GmTsp42El
MGCAATTVKISSIILNLIMAIFAICAIIWISFNPDYIQEQLAIIVYIACSLIVFFAFLGC
FAAVRESMCLMATCAVFLLLLAILQIVFTSNDISEGGMRNGFSIVNEAWESNSMDEIQIE
HECCGKTSANDYVVLSKPIPISCYVDQDYTKVSNLFTEGCSSKLQTYYEDEWWHCSIISW
ILIAFELLAFLLAVFLVINFRNTQRRMRF

>GmTsp42Ei
MGLGTTTIKHILLLLNFVLMFLSLILISFSIFILVSGSANQTDHGENAAGGLIMTLGIVI
LVTAIFGFIAALRESKRKLIIYVPILVMLILTQLVMTSMASHGTRDGLSGSIRQGFEELW
SSEIQAPGALAHYEDWLHCCGVNNTDDYRRIQHEIPKTCCHKRDCSQMENLYQDGCMAKF
EEYLSDKTLAFSIVNCLLIIVEVISAVLGWLMISKLKNEFRRTNARWL

>GmTsp42Ed
MDCGGVLVKYILFVFNILFVVCGLLLIIFGSLMVSNIGEIPSTPESIQANSVPVVILVLG
CIIFVIAFLGCCGAIRENACCTMTYSVIMLTLFICQLALVIFIWQQRVQILDKMDDVIIK
IWDQRHTDQKVMDALQISFKCCGKNGFTDYTLAGEAIPPSCCEVSATSCTAIEAISRPGC
VRAFTNFWENSMNIIRYAGLGVAGVELVAFIFGCCLANQMRNKHRRSHY

>GmTsp2
MNCQTTFYNIVLNIINTVLSLLGVGLIALSVYELNISTPGTFEHIAVIIQIFIGSFLILT
SFLGCFGACRESLGLIWSYGLCVVFLLTFQIYILVVAHTTDYKRNATDDLIKLWQNYPVN
VERIAEVEQNYYCCGKNSTQDYISMGKFIPTSCYQNYERIDSKRYTKSCLEAVQENAAKS
AHIGSSVKWTLFLFEVLALGIASLLGINLRNERRRRLFEN

>GmTsp3
MATIRSYLQWSIVVINTFCVIFGILGVVESALELQKYNYGSLDYTEKVVQIIMGVALVVS
AFIGCGGALNGSLKLLSLYIFVLLMLMIGHVWKLWRYDESRIRNSVEVMITYTWIEELVE
RGKMDALQYSYHCCGKRGYSDYTALSLKIPNSCYQVEKNNKKTFYPYGEGCLSAVSNAYL
DIYRAEKWFHVSLIGVEFFGIVLTVTLICNLTADTLRYSY

>GmTsp4
MGYDCGVWIGKYVLCIFNFLFFVLGTIVLGIGIWLAVDKASLIALLKMVENEHINQFTQP
QVIEQLAYVLIAIGGVMFLMSFMGYCGAIRESKCLLTTYGTFMILLLIAEIVAGGLAAFY
KDTARNETKTFLQSTITKYYTSREHTDAVTLMWNQMMSTFACCGVNDYRDFDMSTAWAAN
KGNRTVPEVCCILKDVKLMVPRDEDCVTNPTEGNSFFKRGCYEVFTEWIVTNRELLVAVL
LSAGFVHLLAIFLAFCLCKSFAKYHGMRL

>GmTsp5
MGFGSRMDCCRQFVKYSLIVSNFIIFVGGATLFCLSLWTLVDRNFMNELLDTNLFSGAVY
VLLVTSMTICVLSFVGCVGASKEVKCLLLTYFIIVVLIFVTMLIGGILGFVFRERVQQTM
RQKLRSSMSLYGSRRDVTESWDQTQERLQCCGVDSWHDWNRLIPESCCQEIFGGQRKECT
LIPAVTNLYSQGCLYVTTSFMRDHAAVIGGTAIAVAIIMIFGMIFSCLLFTMIE

File S1. *Cont.*

```
>GmTsp6
MGLNGCCSCVKFLMILINILFWIIGLTIVIASAWMLTDPTFVLSMTQSYNHYYIALYVFL
GIGVLITVGAFFGCCGVLKESQCLLVSFFCVILIVMVAQIAAGAWAFHNKDKLDDIVRAS
VKYSVQEEYGQSSMSSRTVTFDTIQKNLKCCGADGPSDWATSRFNNVDRTNIVDIAISSM
NVFYNIPESCCKDELKENVCEMSRKLKFGGTFNPAIHQQGCVDKLIELIYENWVLLFGVT
AGVILLELLALTFSLSLCCAVRNHQYKA

>GmTsp7
MGNAGYTCIRRTFCWLNITLWLCSCAFLGAGVWLRLSYEGYATLLPHHAVLSADSMFLGI
GVVGFVISFLGCCGAWVQSKCLLVLYFMLIVLLFVSEFLIGSLAFIFRGGFGRTLANELR
FGIENHYNVSDRGSIIAPSVATIWDNIQISFECCGVSTYEDWYDVQSWSGKRWVPESCCK
SLYENRGILTEGSGDSITKIDCGRSENPSLWWPKGCADSLQTWLNGQLDVIGAVGLGIAF
TQLFGLITSMLLFCTVKHKRKPETYKSYSPSIDPQTRPSSWED

>GmTsp8
MPVRKYRRETTEINCCLKYILFSSNVVIWMTGLVVLAIGIWAWNEKDMFSNITKLTFIAL
DPAFVLICIGGITFTIGFTGSVGALRENTCLLGTYATFLAILLLFEMGFGVLAFVLKDKG
WIKDQATEGLRAFIIHYREDPDQQNLIDWIQEDWLQCCGIEGPKDWDSNNYFNCSSGAIG
SREACGVPFSCCRRRPQELIKNKQCGYDVRKEGYVSDRHTFYYYDWKPVDRHIYERGCLR
AGEDWLESHLIGVSATCIFTLIMQVGNYEVSKIIYEKGCVQAGEEWVERNLIIISISAIV
TVFFQILGICFAQNLRADIYAQKSKWH

>GmTsp9
MSNYRHHSGGGYCGIEIRDMHPRISQNFTYVSSCVKYMIFMLNFIFWLFSGLLLGIGLYA
FMDKWESTGWVRLETVYDVILNISLVMMIAGIVIFVVSFAGCLGALRENTCLLKFYSMCL
LLFFLLEMAIAIMGFVFPQNMNSFLEDKFTDKIIHSYRDDPDLQNFIDFAQKEFKCCGLS
NVGYQDWSKNEYFNCSSPSVEKCGVPYSCCINATDISSGLVNIMCGYGVQEHSVAAASKR
IWTSGCIEIVRVWAERNLYAIAGIALGIALIQLFVIYLAKTLEGQIELQKSRWSA

>GmTsp10
MNSNEILMSYACVRSLLILFNIAFWLSGLSLIWIGVWLQTDFENYLKISENYSDQVHGIL
IIMGAVIIFVTSLACSCIVKIQSSMLAINGGILITMLFSLISLSVYIYTYKDQVMNGIID
GISNEINNYNFSNSTNNDNRDNLLDIVQKNLQCCGNESFTDWPQDAIPKSCFKNFKDYTS
VPDIKNIYETGCAYKLKNVINKNFTAIGVTTSTIALFPFFGAVLSFGLSLMYNKGGYEMI
V

>GmTsp42Eg
MACSTQTLKIFTLLWVFLYTLLASAVIGIGIYIVTKFDHFHTAAIIIIGLGIVALFLAFF
GILSALRENSCLLKTFSAFLIFLTVLLVLTVGFFWIFKTQLFVNVDKTFDKLWREQPVPI
KPGNASQIASIERWLECCGNSGPSDYVLPPYSCYNSQTDKLNMDGCRQKFLDYISERWIA
FNIFALVLAIIELICATFAYVLANSIVNRWRRSKYYPK

>GmTsp39D
MASGLGCIKCITFFCNLLFALTGLLILFVGAMVQLNYSHYSNFVSDQIWTAPIILIIIGS
IVAFICFLGCCGALKENGCMILSFAILAFVIFVCEIGLGIAGYMKHTGLRRIMEAQFNVT
MRDYNDRDDYRDAWSLLQSELECCGTYGPSDWEAVYHNTTLHASCCPIIVLNEADKCTTA
HASPDGCLNKLIHILDSNALVLAGVVLGVASIQLLTILFACCLCRSFCRSYQTV

>GmTsp11
MALTKRIKCFKYLVYCYVVLLTVTGCAQIFIGTSLLWGHSGYYGIVQNKLWAPAAILLCL
GPITFILCWMGCQATNQRKRCLLGMFTTLLVTCICVQFILCGWSLAMRENLPSSVEIFID
DSFVEFLDKFSRTKVDNLHLWNRMQSQLQCCGVDGPLDYRRLSLPWSCCSRPEHAYESAC
DTHYKRGCLAVVSEQIKHRLLITAFSAAIIAIFQSLGLFCAVHLTILFGKSDNGYDNNKL
SKKRQQQFLPLSIQDKRQDMPSPLSLSPTAPGHRVIKTSATGGQK
```

File S1. *Cont.*

>GmTsp29Fb
MALYRDLQLNSGMRCAKYMLLIVSFMFAITAILLVMVGSTIQAIFGDFRQFVDDHFLSPP
ALLIAIGFILLFVATLGAYGAARESVMLINLYGLCLFIVFILEVSASLAAFIMQGQVREM
LIRTMNESLAEYEVNPYIQGGVDFMQSGLECCGVYSPADWSNYWTPNTGNNEEIPVPPSC
CGSFSPDNLMHCEMTYESGCLERMDFIISQSTMLIATGATTVAFVQLLGSICAFMLAKTL
RRNKSIREARRWQLQQSLGVLISGGKMVPPSNSPMTGYTQLEKAEKFSENDPITYTPNSP
SVN

>DmTsp66E
MVFDCGVWCAKYLLCIFNFIFFVLGTIIFGVGLWLAVDKHSLIALLKLVESERIEQFTQPQAIEQLAYVL
LVIGAVMFFMSFLGYLGAMRESRCLLSTYGTFLILLLIAEIVAGGLGAFFKDKVRAESKNFLQTTITSYS
LGENVDATSLMWNQLMGNFGCCGINDYHDFDASPAWVNGKGNRTIPDACCILKDVAKLVPRDEDCTTNPS
DSNSFYKKGCYEVFTEWLIRQRELVIVAIAVGIVHLVLIILAFALCKAFAKYNDMRL

>DmTsp42Eh
MYCTTRLLRYVLCVISGICALGGCLLIWYGAWLLDSLSEEQRMLGMDHGEDLAAVLCVLLGTVIVVASIF
GSVAVAKDSRVLLICYAVLLVFLLIVQIVLVSISYAASRDFLPDSLRQGLDDLWDLQHEGNSTLNTYEEW
LHCCGRNSAEDYLHLEKMPPPSCCLNRDCTKHLNLFMTGCEVKFKEYVGAKTANFHSLSWFLVIFEFAGS
VTTCYLVDSIRNHRDRIRFYN

>DmTsp42Ea
MSCGISMVKYILFIFNLLCSICGILLIVFGALLFSKVRNMDDFAEALRTQQVPVTMIILGTIILLISWFG
CCGAIRESYCMSMTYSILLFVLMIGQLALVIYMWVQKDKYLEIMGDVVEKAWNHRTSRSDYMDAIQISMK
CCGRSGYTDYAYQGKFPPSCCSDTNNCRWETVYRRGCKVTFVEFWDRNSDIIKYAGLVIAAIEFVGFVFA
CCLANSIRNYRRRAEY

>DmTsp97E
MCGGFTCSKNALIALNILYVMIGFLLIGVGVYARAASIVTNLPIVGGILACGVILICISMLGLAGAVKHH
QVMLFFYMIILFMLFLIQFSIASSCLAVNSEQQQQFAEQGWMTVPTDLRKQVQDSLKCCGFNATAPSTTS
VVPPSNEPSCELINQQCCAHSSEPDCRCEPCGPLLEDKIDYAFKLCGGLGIFFSFTEFVGVWLTVRYRNQ
KDPRGLPSAFL

>DmTsp74F
MGFSSRMDCCGQFVKYSLFIANFVIFVGGAIVFCLTLWTLVDRSFVNELLGTNLFSGAVYVLLVTSIIIC
LVSFLGCVGAGKEVKCLLLTYFIIVALVFVTMLIGGVLGYVFRERVQQTMRQEMRSTMALYGSRREITQA
WDLTQERLQCCGVDTWHDWNRYGPVPESCCQELFGGQRKECTIFPTITNLYNQGCLYVTTNFIRDHAAVI
GGTSIAVAILMIFGMIFSCLLFNMIE

>DmTsp68C
MACCFNYKFVLNLCNFLFLICGLLLVVSGLYIFSDNKRILLSRLLAASSDRLSSLPQPLLFYIALGVAIA
GFVATLAAVVGFWASCLHTYCFLTIYFLSVVVLLLTESVLCLAITLWPHCLGISLDETQMVRSLQSNYGV
PGQEQFTNALDLAQVRFGCCGMRSSLDYDTSLWRLQGYGQRNWPVPLSCCFLKNAGHSMAYLDPKPANES
MCQSLERLSYERERHTESCLPHLDNWYREQYSIFLGASLILAMIEFCVLLAIIMSCTGLASQRARLKKPV
QEMRTQKVKSRQTLIENIYEPDVELRENSNHSGDGIYLGPASRHVSSEDFKELYIKPRDLYKQHNLRTSP
ANRPTQMRNYLV

>DmTsp47F
MRSCGPSLIKYVLFAFNVLFAISGLGILIAGAVVLADVNEFNHFVEGRVLAPPIVLIVTGLIIFLIASLG
CFGAIKESPTLLITFAVLLAVIFIVELAVGIAASVFKKDLEGMVKNSLQESIKRSNSEDTMAWDNIQQKL
MCCGVDSPADWRTLSANKTLPGSCCQPQYIDSTVGHCLESPALGKDKYFQVGCVGKLKDRIEKNAIILIG
VGIGIAFIQILGIVLACYLANSIRQERAK

>DmTsp42Eb
MNCLSAMFKYLLYLLNLVFVAGGILLIVVGSIMLSTMGNFTAFDGGVNTQTIPICIIVIGSVTFVVAFFG
CCGTIRENACCTTIYAICMLILFGLQLALSIWIFAANDKFLSSMGKAVDKAWDENNAAQGYPMDALQLAF
SCCGNTGYQQYETVPSSCCGYKDRTKVCEAEIYSQRPGCRQEFVDFWASNTDLIRWSSLIIALFELGIFI
MSCCLASAMRKR

File S1. *Cont.*

```
>DmTsp29Fb
MSNRELQLNSGMKCAKYMLIIVSFMFALTAILLIMVGTTIQTIFGDFSLFIDGHFSSPPALLIAIGFILI
AVAALGAYGAVKESVMVINLYGVCLFLVFILEVSAAIAAFVMQSQVRGMLIRTMNQALAEYEHDPYVESG
VDFMQSMLECCGVNEPEDWKDYLSANVNFTLGVDDVVVPNSCCGNQPTSLNDSTQMTCMETYDYGCFRKM
NFIVSQSAMLIATGATTVAFVQLLGVLCAFMLAKTLRRNKSIREARRWQLQQSLGVLISGGKMAPPQNSA
VTGYQQLDNGEQGSHEPYTYTPQSPSVN

>DmTsp96F
MGLNGCCSCVKYLMVLINILFWLIGLTIVVTSVWMLTDPTFMLSMTQNYNHYHIALYVFLAIGILITLGA
FFGCCGVCRESQCLLVSFFCVILIVMVAQIAAGAWAFHNKDKLDDIVRAAVKSSVQEEYGQSTMSSRTVT
FDTLQKNLKCCGADGPGDWATSRFNNVDRTNIVEIAVSSMNVFYNIPESCCKDNLKDNECELSRRLKFGG
PLNNAIYQQGCVDKLIEIIYENWVTIFAVTAAVILLELLSLTFALSLCCAVRNQHYKA

>DmTsp86D
MSNHRYHQGGNYMHPRISTYPHHFSYVSSCVKYMIFLLNFLFWLFGGLLLAIGVYAFMDKLMDGNGWLRL
DTIYDVIFNISLVMIIAGVIVFTVSFAGCLGALRENTWLLKLYSMCLLLFFILEMSLAIICFVFPQYMNS
FLEYQFTDKIIHSYRDDSDLQNFIDFAQQEFNCCGLSNAGYQDWSKNEYFNCSSPSVERCGVPYSCCINA
TDISSGLVNIMCGYGVQVRSVAAASKRIWTSGCIEIVRVWVERNLYVIAGVALGIALLQLFVIYLAKTLE
GQIDLQKSRWS

>DmTsp66A
MVDNEETGCLQRLLSVGKLQICKFIIYFVWLVNFIFSCADIYIYYFILKEHMPCWDCLFRSYMIIALTVN
ALMAPLLIVGFFFIYSHLCREIRIYATVLFLATWLQMMLTILFAQQYQIVGDVLRIWMNRKSLEFYESRC
CGVLGPDDYKLGDLNIPKSCYKNGSERDEDLYRSGCSTRSIKPASPIIHVISFVIQYVLVICIEVFLIIL
LRSKSQPTSMWSERVTERFGSVKK

>DmTsp42Er
MGWSPLMIRYLAFLFNFLCAVLGIATIVVNVIAIDQIAPKDQLILGLYIAVGSIVFLLSFFGCFGAIKES
ICVTWAYATSMLVMLIVSIVMLFVFRMHFEEDSITKLKQAFAKQTNTFDAMAEYQTQYQCCGIYKLKDYG
DAYITVPSSCYDQNDTPYRDGCLAKMETQYEELLKGPKIVGWMLMVIEIGAFTFSTIMGVSLRNELRRSA
Y

>DmTsp42Eq
MSCGTKALKVSSFVLDFLCCVLAALTIAACSYALIAFSHSVAIRVPSILGIVLGGLLFFSTIFGCIAALR
ESIRMTWIYAAILLALVFSQITVILAQPINYELLANETIYDAWQGQLYHSDRMSYFEIKYHCCGQTGPAN
YPDSGLVIPQSCYFNQNATVTTDLYTVGCNHQLAAAFVKGTRWEKITDWSVVGVEILTVIIAGLLAITLQ
NAERRRLYR

>DmTsp42Ep
MNGCYNTIKYTGLLSNLLYMLLGIGVMSGAGLGLQMAEPNTPEHTYFVKSLVLGGSICMIVMFGCYGMVA
NLLCVNLIFTMFILIALAAEYLQLHHYHSPSLRSPGGAWQQLELAWHGLDRDPELMHQYEASQHCCGYNG
ADDYKRLHLLVPASCYQAAVNDTAQQIYPSGCLETLNRSQRYIQHRDKLYMWAIVGLEIFILLQTVALSV
LLFRLRQRQRIARRQVPPGVRREPRSNHVSASRAHLLNDA

>DmTsp42Eo
MPTVRVCLQWTSVVFSTLTLIVGVLAALAGVYELDKFNEGSAEHTEKFVQLGMAGALILAGLVGCLGAIF
GSIKVMVVNLILLLALIASHIWKVSHYNETKQLDATEVYVMDLWMKELVHHGAMQDLQQEYECCGDKGFS
DYTSLNMKVPRSCFHTKDGIHALYPYGEGCMAAVKRAYLQIYRYEKWVHCGLIGYEVVGIILGITLCCQL
TNKTRRYTY

>DmTsp42En
MDCRTSFLKAVLIVLNVLLSLIGVTLIALSVYELNSSTPGTFEHIAIVVQIFVGTFVVLTSFLGCFATAR
VSLGLVWSYVICLLILLCLQIYIIAAAHSTDYVERSKKDFLATWADQRTNVERISLLEQKYSCCGQLGAH
DYILMGRGIPLSCYKDQERREYSLFSGGCLQAVQAHATDNVAIGLIIKWLLLLVEFAALGAATHLGITVR
NKLRRERF
```

File S1. *Cont.*

>DmTsp42El
MGCATGTIKYSLFLFNALWAILGILVLIFGGLGWGAMPDAYAIGILILGGTILVISLFGCCGAVRESPRM
LWTYASLLLILLLLIVAFIILNPKDVFKKYALQTVENQWELEQTKPGSMDIIQKTYYCCGRDSAQDYLDI
KFWNNTVPSSCCKDDSCVNPLNLYVRGCLIKVEEAFADEATTLGYLEWGLLGFNAVILLLAIILAIHYTN
RRRRYNY

>DmTsp42Ek
MGCTSGCVKCFLNTLNTLNALSGLSLIAIATLALSKAPIAYILFLYGLGGIIFVSAVLGCCGICMENVCM
TATYGFLLLAQLIISLLGIFRFKFTEEYIEKFAAEEVQMKWDEELVEPGAMDIYQTVYECCGRDSPDDYV
AIGRQTLPPSCYPQEDPQMPHYLAGCVQKSSENFVVLFSYAHDTNWIALGITILMMIAAFYLVGRFRKQR
VRYTY
>DmTsp42Ej
MEKSFPITPWKYGLLVTCILIVTCNVFFFSCGVTTWGSAVSVYGSYGSALCGGAVFGVAFLGMYVALKVS
YKYSIYYLICSGLVIAALGSYLFTFTAMREQLMGRFEERMRDLFERKTHSDDKMQPVHSLFGCCGIEGPQ
DYLQEEHGALPSSCCYAFDCSKPAHVYEEGCSTKAVATLRMQAELNYYSCMAIIALEFLGLFTAYHLGKA
RKYAKTKIKDEETPIND

>DmTsp42Ei
MGLGATTVKHVLLLLNFVFSVLGLALIAFGIFFLISAAENAVSIGKNVAGGLIIALGVVILIIAIFGCLA
AIHEAPVRLLIYVGAVVLLILAQLIFLGMSSHGTKDGISGSINEGFDRLWESERNQTGALSYYESWLQCC
GVNSSEDYWIIHHGIPSSCCPESKCMDTPSRVFKTGCKAAFVKYLDDKLLVFKIVCWLLVIGEAVGAVFG
WLLYSSVKNQSRRNNAVWM

>DmTsp42Eg
MACSTNVLKGFALFWDIILALFGLVVIGLGVHIIYKFEHFNTAAFVIIAVGVVVVLTALFGALGAARESS
ATSKVFVVILIVLVILEVLAVGFLWVFQTSLLINVDKTFDKLWNDQPVPIKPGNQSQIASLERWLDCCGN
VGPSDYILPPNSCYNGESDKLNLEGCRQKFLDFIADRWTTFNLVSLVLLGVELICALLAYVLANSIVNRW
RRSKYYQK

>DmTsp42Ef
MASTSSVKLIVYALDVLCTLLALVLISFGIYVAVSYNLNEIGQLTAYGYVGLGAAALLVVLWGYLSAWRE
NVCCTVTFIIFLCLVIIAQFAVVYLLITQEKTVASNLANALEATWEEELNSPGAMSLYQNWFQCCGRGSP
QDYIVNERLPPETCFRNHDKSKPENLIHTGCRVEFENYWQHLTKIFNILALVLIGFELLLSVISCRLCNS
IRNDARRSYF

>DmTsp42Ee
MDCGTSMVKYILFIFNTIVSVIGILGIVYGVLILKSIGVVEVNGQVGFPIQALMPIILISLGSIVVFISF
LGCCGAIRESVCMTMSYATFLLILLILQLTFVVLLFTHREEFENAMGNVIENAWNSEHTYKGGVFDTIQK
SLHCCGSSSALDYIGKGDLVPPSCCSGSCLIPTNYYPGCRGKFVELMTTGSDNAKYVGIGLIGIELIGFI
FACCLANNVRNYKRRNAY

>DmTsp42Ed
MDCGGVFVKYVLFIFNILFVICGILLITFGSIMVSTIKDFSGVGETFTANSVAIIILVLGCVVFLVAFMG
CCGAIRENSCALTSYSVVMLVLLVSQLALIIYVWVDHVQIQQSLEKIVQTIWDQRKTDALLMDTLQRSFK
CCGLNGFADYGITYPASCCDSPSNGTCALTQVMTRSSCLKAVDSFWDTNVSIIKYAGLGVTAVELVAFIF
ACCLANQTRNSQRRQNY

>DmTsp42Ec
MGCLSGIVNFILYIVNIVFLIVGILLIVLGSIMLSDLSRFDVAGSGTDPNTIPICVTVLGGLIFVVSFFG
CYGIFRQSVCMTGAYTSMVFVLFILQLVLTCWVFVNRSAFLGDMSNLVNLLWDSHDYTAMGVLEETFGCC
GDTSYTNYNNIGLSVPGTCCGYLDRQATCNTPSVYQSRPGCSAKFEEFWNDNMDIIRWSGLGLCIFDLVV
FLIAGALTNCMRSQNAGRQVYA

>DmTsp42A
MANPFRWCRVSHDCVFQINVVLVVVGVIFLLDVLSHLYLKAVMFPGLRLYPVALRPWLFWVRALTMVAYI
LNAVLGIHMARQPTVLKYAGYMLVGSVVLLYTISIGVTRFMYRKRFEFFAEMLVLQMWVRDRLGKVEVEF
ECCGRSSVVDYQTASSNRTWPIGSCCGKQNCTGCTAKLSQYLWTIEMDVARDNIIVSVLLFVAMIVMVLH
FKDVQSLDDTSDVDESSELVDDSDAKE

File S1. *Cont.*

>DmTsp39D
MASGGLTCVKYLTFFCNLLFALTGLLIFLVGGMVQLNYAHYSNFVSDHVWTAPIILMIVGAAVAVICFLG
CCGALKESSCMILSFALLAVVIFLFEIGLGLAGYVKHTGLHQIMESQFNSTMQHYKERADYRDAWTLLQT
ELDCCGINGPNDWETVYRNSTLPAACCSVINLSEAKECTNTHATQHGCLQKLLEILDSKTLILASVVLGV
AGIQMLTILFACCLYRSFRRSYDHV

>DmTsp33B
MRQPFRRASVYLHLLLITEAVIGLLILVVTAYYHTVLTGYLSDIECRLVYGYLFGIYVFGAQVVVTFLCS
IAMWRRIWRRRCTPNIRLLLSVWAFYSCVIIASGFGCVWNLYRGVDVLENAADTSLTRGIDMYYSCPEWK
LLWDGLQWHKECCGVHGYKDWMNAEWMPRRENNCTSMVLAPFACCKRSCDSCFNNFLPSEGQSIGGNSRQ
PFPALTVDSINANGCLPAFVSAVWNCFYILMALWVLALKFLIVLCCMTKFIVHRQNEGDGCDNVGLTDDD
GHPLVVVKYPCNVRCVTIAEDDLVSDNVPDINYCNCTEMDDEPCGY
>DmTsp29Fa
MSLLTGSANAVKYTLFGFNLIFLITGIILIAVGAGVGAVYTGYKLFLAGKFFSIPTFLIVIGSFIIIISF
FGCWGALKENYCLVLSFSVMLAIIFILELAAGISGYVLRNDASDLIKTSLTYSLNEYNSINPNATTKLWD
DIQDEFECCGVTSYNDWITAFPNGDLPISCCNVHVGAVGTFTCNNAQSSVADRHKVGCLDGFSGYISAHA
VSLGAAGVVIAILQFFGVIFACYIAREIKIRNGITGFM

>DmTsp26A
MPAAVRKFRRETSEISCCLKYLLFASNVILWLSALLVLSVGIWAWSEKGMFRNIARLHFIALDPAFVLII
LGGVTFLLGFMGSVGALRENTCLLGAYAIFLSVLLIAEIGFCAVAFVLKDKGWIKDQATEGLKAFIRHYR
EDADQQNLIDWIQEDWLQCCGIDGPKDWDSNNYFNCSSIAIGSREACGVPFSCCRRRPQEVIKNKQCGYD
VRKEGYPVDRNIHERGCLRAGEDWLEAHLISVAIGCVALLVLQILGICFAQNLRADIYTQKSKWH

>DmTsp5D
MGNAGYTCIRRTFCWLNIILWLCSCAFLGAGLWLRLSYAGYATLLPQHAGLSADTIFMGIGGTGFVVSFF
GCCGAWVQSRCLLVLYFMLIVMLFMSEFLVGSIAFLFRGGLGRTLANELRFGIERHYNSSDRGSLVAPSV
ASIWDSVQQSFECCGVSSYEDWYDIQSWPGRRWVPESCCRTLYDQRQVLTEGSGDGMMRPDCGRSENPSL
WWDKGCAHSLQSWFTGQLNVVGAVGLGIAFVQLFGLITSMLLFCTVKHKRASDTYKSYSPSIDPQTRTSS
WED

>DmTsp3A
MSNYRYQGAGLGGVGMAGGRGYSGIEVHEVMHPHHFTYVSQCVKYMIFLLNFVFWLFGGLLLGIGVYAFR
DKWEDANGSVRLENFYDVFLNISLVMILAGTVIFLVSFSGCVGALRENTFLLKFYSMCLLLFFLLEMAIA
IVCFVCPQYMNTFLEKQFTHKIIHSYRDDPDLQNFIDFAQQEFKCCGLSNSGYQDWSKNEYFNCSSPSVE
KCGVPYSCCINATDISSGLVNIMCGYGVQNAPVPEATKLIWTSGCIEIVRVWAEHNLYVIAGNALGIALI
QLLVIYLAKTLEGQIELQKSRWLA

>DmTsp2A
MGIGYGASDEQLEKQIGCVKYTLFCFNIVAWMISTALFALTVWLRAEPGFNDWLRILEAQSFYIGVYVLI
GISIVMMAVSFLGCLSALMENTLALFVFVGTQVFGFIAIVAGSAVLLQFSTINSSLQPLLNVSLRGFVAT
SEYTYSNYVLTMIQENIGCCGATGPWDYLDLRQPLPSSCRDTVSGNAFFNGCVDELTWFFEGKTGWIVAL
AMTLGLLNVICAVMSFVLVQAVKKEEEQASNYRR

>DmTM4SF
MALPKKIKCFKYLVYSYVVLLALTGAAQIFLGTSLLWGHSVYYGIVQNKLWAPAAILLCLGPVTFILCWM
GCQATNQRKRCLLGMFAALLVACICVQFIICGWSLAMRENLPTSVEIFIDDSFVEFLDKFSRTKVDNLHL
WNRMQSQLQCCGVDGPLDYRRLSLPWSCCSRPEHAYESACDTHYKRGCLAVVSEQIRNRLLITAFGAAII
AIFQSLGIFCAVHLTILFGKNDNTHPMNMNRKKKQQQFLPLTIQDKRHDMPSPINLSPSAPGQRVLKTAL
PSAMHK

>Dmlbm
MGCATTSVKIASIVLNAVLGFLAAGAIGWIAYNADTETEEFVIAAYIACSLILVFALLGIFAAIRESVVL
TATSAVFLLILAILQIVSTCLFLHEFDVKSGRDMVEVAWQANNMDSLQQKHECCGQSSAQDYIHLSLLIP
PSCYADLQQTPDHLYLDGCIEKVQSFYESDKLRFIIVSWVLVAFELICFALAVFLAISFKNKQRRMEF

File S1. *Cont.*

>DmCG30160
MNCLSAMFKYLLYLLNLVFVAGGILLIVVGSIMLSTMGNFTAFDGGVNTQTIPICIIVIGSVTFVVAFFG
CCGTIRENACCTTIYAICMLILFGLQLALSIWIFAANDKFLSSMGKAVDKAWDENNAAQGYPMDALQLAF
SCCGNTGYQQYETVPSSCCGYKDRTKVCEAEIYSQRPGCRQEFVDFWASNTDLIRWSSLIIALFELGIFI
MSCCLASAMRKR

>Md-T1P8U3
MGLNGCCSCVKFLMVLFNILFWVIGLAIVIASAWLLTDPTFVLSMTQSYNHYYIALYVFL
GIGVLITIGAFFGCCGVLKESQCLLVSFFCVILVVMVAQIAAGAWAFHNKDKLDDIVRAS
VKYSVQEEYGQSSMSSRTVTFDTIQKNLKCCGADGPADWATSRFNNVDRTNIVDIAISSM
NVFYNIPESCCKDELKENVCEMSRKLKFGGTINPAIHQQGCVDKLIEVIYENWVLLFGIT
GGIVLLELLALTLSLSLCCAVRSQQYKA

>Md-T1PBG6
MGCATRTIKCVLLIFNTLWAIFGVLLLLLAGFGWDAMPQNYGIGIIALGCVILLTSLFGC
FGSIRESARSLWTYAVMLLIILVLTVVFICLNTRDVFKNYAIQEVNQLWEQELIHPGAMD
QKQLVYECCGKNGPDDYLLAGRITPSSCCKDSNCINPLNIHINGCLSKIEEAFSNEALTS
QISEWVLLGLNAVVLLMSIMLAIHYTNQRRRFNY

>Md-T1PB83
MDCGTTFAKYVLFIFNAIVSILGILVIVFGVLILNSIGMIEVDGQTGFPPQAAMPIGMIT
IGSIVVFISFLGCCGAIREDVCMTMCYAVLMLILLIIQLIVVVLLWTNQDKIETAMDNVI
ESAWQSEVREAGVFEVVQKSLKCCGVNSAADYALNGRIPPKSCCPADETCIVINYYPGCK
KEARKFITGSSEKAKYFGLGLIVVELVGFIFACCLANNVRNSKRRNAY

>Md-T1PDV0
MACSTKTLKFFSIKWDVIYAILSIALIVLGAFILIKFERFDTVGYTLVGMGSLVLLFTIV
GIVGAARERSTLLKIFALIVVILAITHVIILGFFWIFQTSFLINADKAFDEIWRDQPTPI
KPENGSHIANIERWLSCCGNSGSVDYHLPPHSCYDSKTDKLNTDGCRQKFVDFITESWTY
LNIFVLVYVVIELICAIFAFVLANSIVNRWRRSKYYSK

>Md-T1PAF0
MSCGMTMVKYTLFLFNILCSICGILLIVFGAMLFSNIHTVEDFAEAVKTQQVPLTMIVLG
IVILLISFFGCCGAIRESYCMSMTYSILLFVLMIGQLVLVTYMWLQKDKYLVIMGDVVEK
AWQNRTRKSDYMDALQISMECCGRSGRADYHFQGYYPPSCCKDVRQCNDSTAFNIGCKQA
FVEFWDKNADIIKYAGLAIAAIEFAGFIFACCLANNIRNYKRRSGY

>Md-T1P904
MGFSSRMDCCGQFVKYSLFVSNFVIFVGGATVFCLSLWTLVDRSFMNELLGTNLFSGAVY
VLLVTSVAICLLSFLGCVGAGKEVKCLLLTYFIMVALVFVTMLIGGILGYVFRERVQQTM
RQEMRSSMALYGSRRDITIAWDQTQERLQCCGVDTWHDWNRFGPIPESCCQEIFGGQRKE
CTIFPTITNLYNQGCLYVTSNFIRDHAAVIGGTSIGVAIVMIFGMIFSCLLFNMIE

>Md-T1PJU0
MYISKRTYKIIVIILNVICAILGSVLTWFGSWLYKSIDDEKFDHGEKLASIVIMTLGGVL
IFTAIYGTLATVFELSNMLISFAVLLIALLVMQIVLVSISYTALNNGVSKRLEKGFDELW
DPLHLKPSENLSFYEEWLQCCGKNSPEDYYLLDKYPPTSCCKDHNCIKIHNLYKDGCADK
YRDYVKSKVENFNILSWVIIATEFIGSIFSCLLIDSIRNYRDLRRFYS

>Md-T1PCN8
MGCTSDCMKCILNIFNVLYGISGIVLIVFGVSLLQSANIVQLYIIIAMGAINLIAAILGC
CGICHEHVCMTATYSLFILSSLVGQIYNKIKPASTDEIKKIIRSTIKAVWDEELKTPGAM
DSTQKSLKCCGLDGPTDYQRNGRFVLPASCYPDDVSTAGHAFFNVGCVTASENKVFSLAK
YQTSSEWGVIAVTAVLFIFSIYLVFRFNSRNKRYRY

File S1. *Cont.*

>Md-T1PBY6
MGCATTTVKISSIAFNIILALFAIASIIVISFNPDSMREDIAIGAYIACSLIVFFAFLGC
FAAIRESVCLTATCAVFLLVLAAVQIALTCVGMTDTGVRSAVETVDKAWESNAMDAIQSE
HECCGKISPNDYITLNKAIPISCYIDEDASKTSNLFFEGCKDKLQRHYESEAYIFTVVSW
SLVAFVIIGFLLAVFLAISFRNTQRRMQF

>Md-T1PF15
MKEYNPTHTNDVTFMWDYVQRTFTCCGVESYKDWNTVLNGSLPLSCCRNPVGHVGSFTCT
MNNEDVNRYSLGCLSEFSNYIAAHAVSLGAAGVVIAIIQFFGVLFACYIAREIKIRNGIT
GFMG

>Md-T1PC83
MLIILLFLSEFLIGSIAFVFRGGLKRTLANELRFGIEEHYNATDRGSIIAPSVAAIWDNV
QMSFECCGVSSYEDWYDIKSWSGKRWVPESCCRPIYENRGILIEGSGDSVLRVDCGRSEN
PTLWWDKGCAQSLQTWLTDKLDVIGVVGLGIAFAQLFGLITSMLLFCTVKHKKDSDTYKS
YSPSIDPQTRPSSWED

>Md-T1P9W2
MSNYRHHIGSGGYCGIEVRDMHPRISQNFTYVSSCVKYMIFMLNFIFWLFGGLLLAIGLY
AFKDKWESTGSVRLESFYDVILNISLVLMIAGVVVFVVSFAGCLGALRENTCLLKFYSMC
LLLFFLMEMAIAIMGFVFPQNMNSFLEETFTDKIIHSYRDDPDLQNFIDFAQQEFKCCGL
SNAGYQDWSKNEYFNCSSPSVEKCGVPYSCCINATDISSGLVNIMCGYEVQAHSVAAASK
RIWTSGCIEIVRVWAERNLYAIAGIALGIALIQLFVIYLAKTLEGQIDLQKSRWSA

>Md-T1P8S2
MATRDLQLNSGMRCAKYMLLIVSFMFAITAILLVMVGSTIQAIFGDFRQFVDDHFLSPPA
LLIAIGFILLFVATLGAYGAIKESVMLINLYGVCLFLVFILEVSASIAAFVMQGQVREML
VRTMNESLANYESNEYIQAGVDFMQSGLECCGVDGPRDWINFIQQSNSTDNRIDVPLSCC
GMYSYDLESCEKQYENGCFGRMDFIISQSTMLIATGATTVAFVQLLGALCAFMLAKTLRR
NKSIREARRWQLQQSLGVLISGGKMAPPMNSPMTGYTQLEKSERFYEQEPIAYTPNSPSV
N
>Md-T1PJ11
MLATSLFALTVWLRAEPGFNEWLKILQAEAFYIGVYILIAISIIMMAVSFLGCLSALMEN
TLALFVFIGTQIFGFLISIVGSALLMQYSTMHSSLQPLLQTSLSNFVSTSEQPYSSYVLN
MIQENVGCCGASGPWDYLNLRQPLPSSCRDTVSGNAFFNGCVDELTWFFEAKTSWIVAIA
LGLAMLNVISAVMSLVLVQAVKKEEEEVQAYRR

>Md-T1PD63
MGSDCGVWFGKYVLCIFNFVFFILGTIVLGTGIWLAVDKSSLIALFKMVESEHINEFTQP
QVIEQMAYVLIAIGGFMFLMSFLGYCGAIRESRCLLTTYGVFMILLLIAEIVAGGLAAFY
KETARNESKGFLQSTITKYYTSSEHTDAVTLMWNQMMTTFGCCGVVDYRDFEQSPSWLTS
KGNRTVPEACCRLKDIKNLIPEDEFCVTNPSEANSFYMKGCYETFTDWIIIHREIIIGVL
AGAGIIHLLVIFLAFCLSKSFAKYHGMRL

>Md-T1PC85
MASKSAVKRFIYIFDILCILLASVLIGFGAYVICTNETNEIGTMGAYGYIAIGVATFVVF
IFLNVGAMRDNVCCTVTFIVLMVLIIFAQGVVAFFMIAGRESVASNLANELDATWEKELK
NHGAMSIYENWFDCCGRASPQDYIVAGRLPPPTCFVGHDISLPENLIESGCRIKFEDYWL
ELLGIFNILACVLIGLELILSFIACCLCVSIRNERRRSYY

File S2. Analysis of selection pressures exerted on tetraspanins in *Glossina morsitans, Drosophila melanogaster* and *Musca domestica*: The analyses revealed that several sites are under positive selection based on statistical significance tests as assessed by various models such as MEME, SLAC, FEL, IFEL, REL and FUBAR (Tables S1 to S6, respectively).

Table S1. Codons under positive selection based on MEME analysis.

Codon	α	β^-	*Pr* $[\beta = \beta^-]$	ρ *value*
7	0	0	0.680891	0.000419473
8	1.62595	0.89548	0.929245	0.0749407
10	0	0	0.621438	0.0000006
11	0.915328	0.90513	0.887747	0.0228057
12	0	0	0.967885	0.000061
13	0	0	0.925898	0.0537399
15	0	0	0.798366	0.000000022
16	0	0	0.944415	0.00271562
22	0	0	0.627583	0.000637962
25	0	0	0.673026	0.00850381
30	0.22188	0.22188	0.687667	0.0000029
33	0.112483	0.112483	0.707372	0.000007544
35	0	0	0.672794	0.00004139
37	0.521743	0	0.510877	0.0186541
38	0.119991	0.119991	0.715983	0.0000000149
39	0.00118	0.001178	0.625878	0.000346185
41	0.07541	0.07541	0.84294	0.0000000014
42	0.08907	0.08907	0.715871	0.0164398

Table S2. Codons under positive selection based on SLAC analysis.

Codon	*dN/dS*	*Normalized dN/dS*
3	12.7691	0.6068
7	7.51301	0.3570
9	5.82693	0.2769
10	13.4225	0.6379
11	16.4621	0.7823
14	12.0891	0.5745
17	11.6889	0.5555
23	8.64387	0.4108
26	8.54061	0.4059
28	10.7545	0.5110
32	14.9546	0.7106
33	9.72562	0.4622
37	9.21561	0.4379
38	10.1208	0.4810
40	10.2132	0.4854
41	6.8901	0.3274
42	5.82768	0.2769

Table S3. Codons under positive selection based on FEL analysis.

Codon	*dN/dS*	*Normalized dN/dS*
7	3.435	0.0651972
10	10.715	0.0645841
15	3.179	0.0446692
22	4.311	0.0849642
39	5.425	0.0498576
41	14.162	0.0629439
42	3.133	0.0375647

Table S4. Codons under positive selection based on IFEL analysis.

Codon	*dN/dS*	*Normalized dN/dS*
7	4.547	0.1031
10	9.872	0.0544
15	4.709	0.0720
22	4.292	0.0844
25	2.949	0.0627
39	6.271	0.0555
41	14.444	0.0653

Table S5. Codons under positive selection based on REL analysis.

Codon	*Normalized E[dN-dS]*	*Posterior Probability*
6	0.0134554	0.993061
10	0.551239	0.998573
11	0.643135	1
15	0.769529	1
16	0.55005	1
22	0.0236506	0.992037
25	0.545118	0.999993
31	0.546731	0.999663
38	0.545081	1
39	0.540439	0.993907
39	0.608109	1
40	0.540071	0.997656
41	0.732863	1
42	0.554127	0.999987

Table S6. Codons under positive selection based on FUBAR analysis.

Codon	***Β-α***	***Prob β > α***
10	0.168443	0.998531
15	0.110881	0.965741
22	0.412523	0.997355
38	0.425619	0.998779
39	0.117648	0.946172
40	0.290936	0.945438
41	0.167577	0.99861
42	0.0886742	0.90174
